# Peripheral arterial disease progression and ankle brachial index: a cohort study with newly diagnosed patients with type 2 diabetes

**DOI:** 10.1186/s12872-022-02722-6

**Published:** 2022-06-27

**Authors:** João Soares Felício, Franciane Trindade Cunha de Melo, Giovana Miranda Vieira, Vitória Teixeira de Aquino, Fernanda de Souza Parente, Wanderson Maia da Silva, Nivin Mazen Said, Emanuele Rocha da Silva, Ana Carolina Contente Braga de Souza, Maria Clara Neres Iunes de Oliveira, Gabriela Nascimento de Lemos, Ícaro José Araújo de Souza, Angélica Leite de Alcântara, Lorena Vilhena de Moraes, João Felício Abrahão Neto, Natércia Neves Marques de Queiroz, Neyla Arroyo Lara Mourão, Pedro Paulo Freire Piani, Melissa de Sá Oliveira dos Reis, Karem Mileo Felício

**Affiliations:** Endocrinology Division, University Hospital João de Barros Barreto, Federal University of Pará, Mundurucus Street, 4487, Guamá, Belém, Pará 66073-000 Brazil

**Keywords:** Ankle-brachial index, Peripheral arterial disease, Type 2 diabetes mellitus, Drug-naive, Diabetes mellitus

## Abstract

**Background:**

Little is known about the evolution of peripheral arterial disease (PAD) since diagnosis and its association with glycemic and lipid control in patients with Type 2 Diabetes Mellitus (T2DM).

**Objective:**

Evaluate the actual criteria to start screening PAD with ankle-brachial index (ABI) in T2DM patients and assess its progression and relationship with glycemic and lipid control since diagnosis.

**Methods:**

We performed a 3-year prospective cohort study with two groups: group 1 (978 individuals with T2DM undergoing drug treatment) and group 2 [221 newly diagnosed drug-naive (< 3 months) patients with T2DM]. PAD diagnosis was by ABI ≤ 0.90, regardless any symptoms.

**Results:**

As expected, abnormal ABI prevalence was higher in group 1 vs. Group 2 (87% vs. 60%, *p* < 0.001). However, abnormal ABI prevalence did not differ between patients over and under 50 years in both groups. Our drug-naive group stabilizes ABI (0.9 ± 0.1 vs 0.9 ± 0.1, *p* = NS) and improved glycemic and lipid control during follow-up [glycated hemoglobin (HbA1c) = 8.9 ± 2.1 vs 8.4 ± 2.3%, *p* < 0.05; LDL = 132 ± 45 vs 113 ± 38 mg/dL, *p* < 0.01, respectively]. When compared, patients who evolved with normalization or maintained normal ABI levels at the end [Group A, *N* = 60 (42%)] with those who decreased ABI to abnormal levels (ABI basal 1.0 ± 0.1 vs final 0.85 ± 0.1, *p* < 0.001) [Group B, *N* = 26 (18%)], an improvement in HbA1c (9 ± 2 vs 8 ± 2%, *p* < 0.05) and a correlation between the final HbA1c with ABI (*r* = − 0.3, *p* = 0.01) was found only in the first. In addition, a correlation was found between albuminuria variation and ABI solely in group A (*r* = − 0.3; *p* < 0.05).

**Conclusion:**

Our study suggests that ABI should be measured at diagnosis in T2DM patients, indicating that current criteria to select patients to screen PAD with ABI must be simplified. An improvement in albuminuria and glycemic and lipid control could be related with ABI normalization in newly diagnosed T2DM drug-naive patients.

## Introduction

Cardiovascular diseases are the main cause of mortality among patients with diabetes [1, 2]. Peripheral arterial disease (PAD) is an important member of this group and is associated with lower limb amputation and potentially fatal outcomes, such as acute myocardial infarction and stroke [3, 4]. Therefore, diabetes mellitus (DM) is a strong factor associated with an elevated risk of PAD: the prevalence is almost 2–4 times higher in patients with diabetes when compared to the general population, affecting 20–30% of people with diabetes [5, 6].

Moreover, PAD’s clinical presentation is variable, over 50% of patients are asymptomatic and remain undiagnosed, even though this group benefits the most from proper treatment and risk factor modification [7].

ABI is a non-invasive, low cost and easy to perform test used for the identification and determination of PAD severity. It corresponds to the ratio between the systolic blood pressure of the ankle and the arm. ABI has been considered a viable test to detect PAD, its diagnostic sensitivity ranges from 29 to 95% (average = 63%) and its specificity varies from 58 to 97 (average = 93%) [4]. In a recent meta-analysis, Chuter et al. reported sensitivity and specificity of 60% and 87%, respectively, for ABI in patients with diabetes [8].

The strategy to perform ABI test varies among medical entities and scientific works. According to the recommendations of the American College of Cardiology (ACC)/American Heart Association (AHA), DM patients > 50 years or < 50 years with additional risk factors for cardiovascular disease should be screened for PAD [9]. Otherwise, the American Diabetes Association (ADA) recommends that ABI test should be performed only in Type 2 DM (T2DM) patients with signs or symptoms of PAD [10]. Nevertheless, previous data from our cohort group suggested that, in T2DM, ankle-brachial index should be measured at diagnosis [11].

There are few data suggesting that lipidic and glycemic control could reduce complications and progression of PAD [12–16]. It was described in T2DM patients (not drug-naive) in the UKPDS study, which presented that a reduction of 1% in glycated hemoglobin (HbA1c) was associated with a 43% decrease in the risk of amputation or death [14]. In addition, Kim et al. in a cohort study with a 6-year follow-up of 194 newly diagnosed T2DM patients found that the earlier achievement of glycemic control with HbA1c < 7% led to a lower frequency of macrovascular complications [17]. In fact, T2DM cohort groups with drug-naive patients followed since diagnosis are necessary to clarify this issue.

## Methods

### Study design and patients

We performed a prospective cohort study to evaluate Peripheral Arterial Disease (PAD) assessed by ABI in patients with Type 2 Diabetes Mellitus (T2DM) between January 2016 and December 2020. This study was approved by the University Hospital João de Barros Barreto ethics committee in accordance with the standards of the National Health Council. Informed signed consent to participate was developed according to the Declaration of Helsinki and the Nuremberg Code and was approved by the University Hospital João de Barros Barreto ethics committee. Written informed consent was obtained from all patients included in the study. All procedures followed were in accordance with the ethical standards of the responsible committee on human experimentation (institutional and national) and with the Helsinki Declaration of 1975, as revised in 2008.

We performed a 3-year prospective cohort study with two groups: group 1 (978 individuals with T2DM undergoing drug treatment) and group 2 [221 newly diagnosed (< 3 months) patients with T2DM who still were drug-naive, considered to be the ones that have received no previous therapy with oral hypoglycemic agents or insulin]. A control group (group 3) with 50 patients without diabetes was evaluated cross-sectionally.

Drug-naive patients with diabetes are considered a rare cohort, which explains the small sample representing this group. In addition, the control group was used as a parameter for the normal decrease in ABI with age and was only used to compare ABI with T2DM patients. Peripheral arterial disease (PAD) was diagnosed when ABI was ≤ 0.90 [18–20]. No clinical symptoms were necessary to establish PAD. Our public Hospital is the main tertiary center for the treatment of endocrinology and vascular diseases in the North Brazil region. Therefore, patients with diabetes in this center often present diabetes complications, including PAD. It is the reason why our PAD prevalence is expected to be very high. Nevertheless, this makes possible the access to a large number of patients with both T2DM and PAD.

Inclusion criteria consisted in: a) patients of both genders, over 18 years of age, diagnosed with T2DM; b) with or without previous treatment for T2DM, that consisted of oral hypoglycemic agents or insulin; c) All must have been followed at the endocrinology outpatient clinic and be willing to attend scheduled appointments. Patients that underwent peripheral revascularization surgery, ABI higher than 1.4, acute ischemia breastfeeding or pregnant women, or those patients with comorbidities that could interfere with life expectancy were excluded.

### Clinical and laboratory data

Data was obtained through physical examination and complementary exams. Information on demographics, physical measures (weight, height, blood pressure, and heart rate), cardiovascular risk factors, pre-existing clinical conditions, duration of T2DM (in years), medications in use, laboratory tests, ABI, and body mass index (BMI) was collected and analyzed.

The cardiovascular risk factors assessed were obesity and overweight, dyslipidemia, systemic arterial hypertension, smoking, diabetes duration, and sedentary lifestyle. All parameters were based on ADA’s recommendations for T2DM patients. Sedentary lifestyle was defined as performing less than 150 min per week of physical activity, according to the American Diabetes Association [10]. The clinical conditions evaluated and adjudicated were acute myocardial infarction, stroke, cardiac catheterization, coronary revascularization [percutaneous coronary intervention, and coronary artery bypass grafting], heart failure, angina pectoris and atrial fibrillation.

ABI test was performed based on the American College of Cardiology/AHA recommendations [9]. It was done by the same professional in all patients. To measure the ABI, a portable vascular Doppler, a manual pressure gauge, and a conductive gel are required. The measurements were with the patient in supine position on the stretcher, by measuring the blood systolic pressure in both arms. Then, the systolic pressure was measured in the ankles with a vascular Doppler and a transducer positioned at the level of the dorsalis pedis and over the posterior tibial artery. With a sphygmomanometer, the systolic pressure in both of patient's ankles was checked. The ABI was calculated for each of the lower limbs, using the highest value found in each ankle as a numerator and the highest value found in brachial measurements as a common denominator [21, 22]. ABI was performed by the same operator during all study to reduce variability.

Glycated hemoglobin (HbA1c) and fasting glycemia were measured by the high-performance liquid chromatography method. Serum triglycerides, total cholesterol, low-density lipoprotein cholesterol (LDL-C), and high-density lipoprotein cholesterol (HDL-C) were measured, and serum creatinine was collected and used to calculate the glomerular filtration rate (GFR) through the simplified Modification of Diet in Renal Disease study equation [23]. Ultrasensitive C-reactive protein was measured by the nephelometry method, with a detection limit of 0.01 mg/dL. To evaluate nephropathy presence and stage, albuminuria/creatinine (three isolated urinary samples) were studied by immunoturbidimetry.

After first evaluation, patients were followed for 3 years with standard care visits every three months to register the occurrence of cardiovascular events. Laboratory exams were collected every 3 months and ABI was measured annually. We considered only cardiovascular events adjudicated and confirmed by the study team.

### Statistical analysis

An ABI score was established to evaluate the severity of the arterial obstruction, in which normal individuals were assigned a number as 0—normal, 1—mild, 2 – moderate, and 3—severe obstruction, according to their ankle-brachial index value > 0.9; ≤ 0.9; ≤ 0.7; ≤ 0.4, respectively [9]. To establish the relationship between risk factors, linear and logistic regression models were created. After dividing the groups, the variables between 2 groups that had a normal distribution were compared using the Student's t-test, and for those with a non-normal distribution, the Mann–Whitney test was used. For the comparison between more than two groups between normal and non-normal variables, the One-Way ANOVA and ANOVA on Ranks tests were used, respectively. The Chi-square test was used in the analysis of categorical variables, while to establish correlations between variables Pearson’s or Spearman’s test was used. Receiver operating characteristic (ROC) curve was used to assess the sensitivity and specificity of age as a variable for PAD diagnosis. The cut-off point was defined based on Youden Index (J), with maximum sensitivity and specificity in the ROC curve defined as the minimum value in the equation √ [(1 – sensitivity)2 + (1 – specificity)2], and the accuracy was estimated based on the area under the ROC curve. Statistical significance was defined as a p-value of < 0.05All calculations were performed with SPSS Statistics® 22.0 (IBM Corp., Armonk, NY, EUA) e SigmaPlot 10.0 (SYSTAT SOFTWARE Inc, San Jose, CA, EUA).

## Results

In group 1, 847/978 (87%) individuals presented abnormal ABI and 131 (13%) had normal ABI. Among the patients with abnormal ABI in group 1, 742/847 (88%) were symptomatic [claudication, ischemic rest pain, and tissue loss (ulcer/gangrene)] and 105 (12%) had no symptoms. In group 2, 133/221 (60%) had abnormal ABI and 88 (40%) had normal ABI. Among patients with abnormal ABI in group 2, 80/133 (60%) were symptomatic, while 53 (40%) were asymptomatic. Clinical and laboratory characteristics of both groups are described in Tables 1 and 2, respectively.

**Table 1:** Clinical Features and cardiovascular risk factors at the beginning of the study

Clinical features	Group 1 T2DM	Group 2 T2DM drug-naive	Group 3 Control	*p*
	*N* = 978	*N* = 221	*N* = 50	
Age (years)	60 ± 9	52 ± 10	57 ± 10	< 0.001^ac^
Gender (M/F%)	40% / 60%	44% / 56%	36% / 64%	0.4
BMI (kg/m^2^)	30 ± 5	31 ± 5	29 ± 5	0.1
History of Dyslipidemia (yes)%	90%	67%	34%	< 0.00001*
History of Hypertension (yes)%	75%	40%	42%	< 0.00001^ab^
Sedentary lifestyle (yes)%	42%	19%	58%	< 0.00001*
Smoking (yes)%	53%	43%	34%	0.001^ab^

*T2DM* Type 2 diabetes *mellitus,* *BMI* Body Mass Index, *Group 1* Individuals with T2DM undergoing drug treatment, *Group 2* Newly diagnosed drug-naive (< 3 months) patients with T2DM. *Group 3* Patients without diabetes. *All groups differ between themselves. ^a^ Group 1 vs 2. ^b^ Group 1 vs 3 ^c^ Group 2 vs 3. Statistical significance was set at *p* < 0.05

**Table 2:** Laboratorial data from non-drug naive and drug-naive T2DM patients at the beginning of the study

	Group 1 (non-drug naive)	Group 2 (drug-naive)	*p*
	*N* = 978	*N* = 221	
Glycemia (mg/dL)	174 ± 56	187 ± 65	< 0.05
Glycated hemoglobin (%)	8.6 ± 1.6	8.9 ± 2.1	< 0.001
Glomerular filtration ratio (mL/min)	84 ± 24	106 ± 34	< 0.001
Albuminuria (mg/g)	168 ± 170	38 ± 101	< 0.001
Total cholesterol (mg/dL)	198 ± 48	212 ± 55	< 0.001
Triglycerides (mg/dL)	207 ± 115	222 ± 154	0.8
HDL-C(mg/dL)	43 ± 11	41 ± 9	< 0.05
LDL-C (mg/dL)	115 ± 41	130 ± 42	< 0.001

*T2DM* Type 2 diabetes mellitus, *NS* Not significant, *LDL-C* Low-density lipoprotein cholesterol, *HDL-C* High-density lipoprotein cholesterol. *Group 1* Individuals with T2DM undergoing drug treatment, *Group 2* Newly diagnosed drug-naive (< 3 months) patients with T2DM. Statistical significance was set at *p* < 0.05

Tables 3 and 4 show, respectively, the prevalence of cardiovascular events and a comparison of ABI values between groups.

**Table 3:** Prevalence of cardiovascular events in non-drug naive and drug-naive T2DM patients at the beginning of the study

Cardiovascular events	Group 1 (non-drug naive)	Group 2 (drug-naive)	*p*
	*N* = 978	*N* = 221	
Acute myocardial infarction (yes/no)	17/961 (1.4%)	0/221 (0%)	0.2
Stroke (yes/no)	37/941 (3.7%)	4/217 (1.8%)	0.1
Cardiac catheterization (yes/no)	39/939 (4.0%)	1/220 (0.4%)	< 0.01
Coronary revascularization (yes/no)	32/946 (3.9%)	0/221(0%)	< 0.05
Heart failure (yes/no)	29/949 (2.9%)	1/220 (0.4%)	< 0.05
Angina pectoris (yes/no)	48/930 (4.9%)	0/221 (0%)	< 0.01
Atrial fibrillation (yes/no)	2/976 (0.2%)	0/221 (0%)	0.5

*Group 1* Individuals with T2DM undergoing drug treatment, *Group 2* Newly diagnosed drug-naive (< 3 months) patients with T2DM. Statistical significance was set at *p* < 0.05

**Table 4:** ABI values and peripheral arterial disease (PAD) prevalence at the beginning of the study

ABI	Group 1	Group 2	Group 3	*p*
	(*N* = 978)	(*N* = 221)	(*N* = 50)	
Right ABI	0.86 ± 0.1	0.94 ± 0.2	1.06 ± 0.1	< 0.001*
Left ABI	0.85 ± 0.3	0.91 ± 0.1	1.05 ± 0.1	< 0.005*
PAD (%)	87%	60%	10%	< 0.00001*

*ABI* Ankle-Brachial Index, *PAD* Peripheral Arterial Disease, *T2DM* Type 2 diabetes *mellitus*. *Group 1* Individuals with T2DM undergoing drug treatment, *Group 2* Newly diagnosed drug-naive (< 3 months) patients with T2DM. Group 3: Patients without diabetes. ^*^All groups differ between themselves. Statistical significance was set at *p* < 0.05

ABI levels and ABI score correlated with the occurrence of cardiovascular events (*r* = − 0.1 e *p* < 0.0001; *r* = 0.1 e *p* < 0.001. respectively). Correlations between the presence of abnormal ABI, ABI levels, ABI score and their risk factors are shown respectively in Tables 5 and 6.

**Table 5:** Correlations between PAD presence and its risk factors

PAD Risk factors	*r*	valor-*p*
Age (years)	0.1	< 0.0001
T2DM duration (years)	0.1	< 0.0001
T2DM over 10 years (yes/no)	0.1	< 0.0001
History of dyslipidemia (yes/no)	0.1	< 0.0001
Systolic blood pressure (mmHg)	0.1	< 0.05
History of hypertension (yes/no)	0.1	< 0.0001
Heart failure (yes/no)	0.1	0.009
Sedentarism (yes/no)	0.1	< 0.0001

*PAD* Peripheral Arterial Disease, *T2DM* Type 2 diabetes mellitus. Statistical significance was set at *p* < 0.05

**Table 6:** Correlations between ABI value, ABI score and PAD risk factors

PAD risk factors	ABI Score	ABI Values		
	*r*	*p*	*R*	*p*
Age (years)	0.2	< 0.0001	− 0.1	< 0.0001
Smoking (yes/no)	0.1	< 0.05	− 0.1	0.01
History of Dyslipidemia (yes/no)	0.1	< 0.05	− 0.1	0.01
History of Hypertension (yes/no)	0.1	< 0.0001	− 0.1	< 0.0001
Sedentarism (yes/no)	0.1	< 0.0001	− 0.1	< 0.0001
T2DM duration (years)	0.1	< 0.0001	− 0.1	< 0.0001
T2DM duration over 10 years (yes/no)	0.1	< 0.0001	− 0.1	< 0.0001
Systolic blood pressure (mmHg)	0.1	< 0.0001	− 0.1	0.001

*ABI* Ankle-Brachial Index, *PAD* Peripheral Arterial Disease, *T2DM* Type 2 diabetes *mellitus*. Statistical significance was set at *p* < 0.05

We developed a forward stepwise regression model using ABI value as the dependent variable and the following as the independent ones: age, systolic blood pressure, smoking, BMI, sedentary lifestyle, T2DM duration and dyslipidemia. Only duration of T2DM (*r*^2^ = 0.04) and sedentary lifestyle (*r*^2^ = 0.012) were selected for the model. Additionally, we obtained an inclination coefficient of -0.003 using ABI values and duration of T2DM as variables in a simple linear regression model according to the formula “ABI = 0.976—(0.003 × T2DM duration in years)”, suggesting that, for each year of T2DM duration, ABI levels would lower by 0.003.

A binary logistic regression model was performed to understand the effect of T2DM duration on ABI. The model was statistically significant, *x*^2^ (2) = 17.117, *p* < 0.001, explaining 3.2% (Nagelkerke *R*^2^) of variation and suggests that individuals with 10 years or more of DM have 2.3 higher risk of developing abnormal ABI (OR = 2.29; 95% IC = 1.52—3.45). Using sedentary lifestyle as a dependent variable, another model was created, which was statistically significant, *x*^2^ (2) = 13.946, p =  < 0.001. The model explains 2.8% (Nagelkerke *R*^2^) of the variation and assigns twice the risk for abnormal ABI for sedentary individuals (OR = 1.98; 95% IC = 1.38—2.83).

In group 1, 24% (236/978) patients were asymptomatic. Among them, the prevalence of abnormal ABI was 46% (89/193) in those with 50 years and over and 37% (16/43) in those with less than 50 years (NS). Additionally, in group 2, the prevalence of abnormal ABI also did not differ between the patients over and under 50 years [60% (55/91) vs 60% (78/130), NS], respectively. Furthermore, 24% (53/221) of patients in group 2 presented abnormal ABI without any symptoms. When compared with group 2 patients without abnormal ABI, age differences were not observed (52 ± 10 vs 53 ± 11, *p* = NS), suggesting that age would not be a good criterion to indicate the screening of abnormal ABI in asymptomatic T2DM patients.

The prevalence of at least one additional risk factor for atherosclerosis (dyslipidemia, hypertension, smoking, or obesity), when considered all T2DM patients, was 97% (1163/1199). When patients in group 1 and 2 with abnormal ABI were evaluated separately, the prevalence of at least one additional risk factor for atherosclerosis were 99% (840/847) and 87% (116/133), respectively. In patients without abnormal ABI, the presence of one additional risk factor was 98% (129/131) in group 1 and 90% (79/88) in group 2, It suggests that the presence of atherosclerosis’s risk factors also should not be used as a criterion to indicate PAD screening in those groups.

We evaluated the presence or not of a cut-off age in which there would be a significant increase in PAD prevalence. For this, we tested all ages, from year-to-year, between 40 and 55 years and it was not possible to establish a threshold.

Finally, we performed a ROC curve using age as variable for PAD diagnosis, and the value > 55 years was found as a cut-off point with better accuracy, however, the Area Under the Curve (AUC) indicated low precision (Sensitivity: 65.8%; Specificity: 50.2%; AUC: 0.598; *p* < 0.001). This suggests the absence of an adequate cut-off point for the age group concerning PAD prevalence, despite the expected increase in prevalence over time.

A re-evaluation of drug-naive patients after 3 years is shown in Table 7. Until now, 143 patients in the drug-naive group have completed the follow-up period and were reevaluated. There was an increase in the prevalence of sedentary lifestyle over time (23% vs 50%, *p* < 0.001), however, there was no change in BMI (31 ± 5 vs 31 ± 5, *p* = NS) nor in systolic arterial pressure levels (136 ± 22 mmHg vs 134 ± 20 mmHg, *p* = NS) and diastolic arterial pressure levels (81 ± 13 mmHg vs 82 ± 13 mmHg, *p* = NS). The use of oral antidiabetic medications and insulin were observed in 86% and 14% of these patients, respectively, and 69% were in use of statin. There were improvements on glycemic and lipids control and reduction of urinary albumin excretion (Table 7).

**Table 7:** Drug-naive patients’ laboratory features before and after follow-up

Laboratory features	Before	After	*P*
	*N* = 143	*N* = 143	
Glycemia (mg/dL)	187 ± 63	170 ± 65	< 0.05
Glycated hemoglobin (%)	8.9 ± 2.1	8.4 ± 2.3	< 0.05
Creatinine (mg/dL)	0.8 ± 0.2	0.9 ± 0.3	< 0.001
Glomerular Filtration Ratio (mL/min)	104 ± 36	95 ± 25	< 0.001
Urinary Albumin excretion (mg/g—Log10)	1.14 ± 0.6	0.95 ± 0.7	< 0.001
Total cholesterol (mg/dL)	212 ± 56	199 ± 57	< 0.05
Triglycerides (mg/dL)	222 ± 158	228 ± 266	0.8
HDL-c (mg/dL)	40 ± 7	43 ± 11	< 0.001
LDL-c (mg/dL)	132 ± 45	113 ± 38	< 0.001

*LDL-C* Low-density lipoprotein cholesterol, *HDL-C* High-density lipoprotein cholesterol. Statistical significance was set at *p* < 0.05

When patients were re-evaluated, there were no changes in ABI values (0.9 ± 0.1 vs 0.9 ± 0.1, *p* = NS) or PAD prevalence (56% vs 58%, *p* = NS). Between patients with abnormal ABI at baseline (80/143), 23 (29%) evolved with ABI values normalization, in these patients the ABI value increased from 0.82 ± 0.1 to 0.98 ± 0.1, *p* < 0.001. In addition, an increase in statin use occurred during the follow-up (50% vs 69%, *p* < 0.001).

Table 8 compares patients who had normal ABI by the time of reassessment (patients who evolved with normalization or who maintained normal ABI values, Group A, *N* = 60) with those who developed abnormal ABI (ABI basal 1.0 ± 0.1 vs final 0.85 ± 0.1, *p* < 0.001) during the follow-up (Group B, *N* = 26). An improvement in HbA1c (9 ± 2 vs 8 ± 2, *p* < 0.05) was observed solely in Group A.

**Table 8:** Comparison between patients with normal ABI at the moment of re-evaluation (Group A) vs patients who developed PAD (Group B)

Feature	Group A (*N* = 60)		Group B (*N* = 26)		P
	Before	After	Before	After	
HbA1c (%)	9 ± 2	8 ± 2	9 ± 2	9 ± 2	0.05^a^
Glomerular Filtration Ratio (mL/min)	107 ± 23	100 ± 24	100 ± 32	92 ± 24	< 0.05^ab^
Albuminuria (mg/g—Log10)	1.1 ± 0.6	0.9 ± 0.7	1.2 ± 0.6	0.9 ± 0.6	< 0.05^ab^
Total cholesterol (mg/dL)	203 ± 59	192 ± 60	224 ± 56	205 ± 61	< 0.05^b^
HDL-c (mg/dL)	39 ± 6	42 ± 9	43 ± 8	46 ± 10	< 0.05^ac^
LDL-c (mg/dL)	125 ± 44	104 ± 33	142 ± 51	123 ± 38	< 0.05^abd^

*LDL-C* Low-density lipoprotein cholesterol, *HDL-C* High-density lipoprotein cholesterol. *a* = group A differs before vs after; *b* = group B differs before vs after; *c* = group A and group B differ before vs before; *d* = group A and group B differ after vs after. Statistical significance was set at *p* < 0.05

During the re-evaluation of the 143 patients, we found a correlation between the variation in ABI and the variation in albuminuria (*r* = − 0.2; *p* < 0.05), suggesting that the reduction in urinary albumin excretion could be associated with an improvement in ABI. Additionally, in group A (patients who evolved with normalization or who maintained normal ABI levels) a correlation was found between the final glycated hemoglobin with ABI (*r* = − 0.3, *p* = 0.01) and also with the variation in albuminuria during follow-up (*r* = − 0.3; *p* < 0.05). Finally, when we analyzed only the 23 patients who had abnormal ABI at baseline and normalized ABI at the end of the study, we found a correlation between the final ABI and the variation in glycated hemoglobin (*r* = − 0.5; *p* = 0,01).

## Discussion

Our study followed drug-naive patients since the diagnosis of T2DM and associated better glycemic control and reduction of albuminuria with ABI normalization. Furthermore, our data reinforce our group's previous findings about the need to re-evaluate the current criteria for screening PAD in T2DM patients, suggesting that it should happen at the moment of diagnosis. Additionally, sedentary lifestyle and diabetes duration were the risk factors most related to the presence and severity of PAD.

Over a 3-year period, we observed 221 individuals with T2DM – not only newly diagnosed but also drug-naive – with a high prevalence of PAD (60%) assessed by ABI at the moment of diagnosis. It could be, at least in part, to the fact that our hospital is the state reference to vascular diseases. In 2005, Faglia et al. showed, in a study with this subgroup of patients, a prevalence of 21.1% of PAD represented by ABI < 0.9 [24]. Recently, Khalil et al. (2018), in a cross-sectional study evaluating chronic complications in T2DM patients, reported that there was a considerable proportion of PAD in 183 patients newly diagnosed with T2DM [25]. This evidence may be due to the delay in diagnosis, generally caused by asymptomatic or oligosymptomatic presentation of T2DM [26]. Our ROC curve that used age as a variable for PAD diagnosis indicated low precision. In addition, the prevalence of at least one additional risk factor for atherosclerosis did not differ between patients with or without abnormal ABI, symptomatic or not. Thus, our data suggests the implementation of a diagnostic strategy at the moment of diagnosis for this condition as universal screening in all newly diagnosed T2DM patients, regardless of risk factors, symptoms, and age.

Our logistic regression models showed that sedentary lifestyle doubles the risk of PAD (assessed by ABI) and individuals with diabetes for more than 10 years have a 2.3 times greater risk of developing this disease. Similar to our findings, previous studies showed the association between a sedentary lifestyle and low ABI values, both in patients with and without diabetes [27, 28]. Wilson et al., analyzing lifetime cumulative exposure to physical activity in a population at high risk for atherosclerosis, showed that low physical activity is associated with reduced levels of ABI [29]. A study by Parsons et al., evaluating physical activity and sedentary behavior, demonstrated that higher physical activity and lower levels of sedentary behavior were also associated with a lower risk of PAD [30]. Parmenter; Dieberg and Smart, in a meta-analysis, also found a strong relationship between regular exercise and PAD [31]. They showed that even with a significant improvement in cardiorespiratory function and the ability to walk short distances, prognostic and diagnostic criteria such as ABI, blood pressure index, and flow-mediated dilation remain unchanged after an exercise period, suggesting that changes in blood flow or pressure should not be the mechanism by which physical activity improves the ability to walk in this population [31]. Otherwise, Barone Gibbs et al., in a randomized study evaluating T2DM without PAD, showed an improvement in the ABI, suggesting a possible preventive effect of the practice of physical activity [32]. If it is true, the effect of physical activity on T2DM peripheral circulation is higher if initiated earlier.

ABI has been considered a viable test to detect PAD, its diagnostic sensitivity ranges from 29 to 95% (average = 63%) and its specificity varies from 58 to 97 (average = 93%) [4]. In a recent meta-analysis, Chuter et al. reported sensitivity and specificity of 60% and 87%, respectively, for ABI in patients with diabetes [8]. It is influenced by several factors, decreasing the test's sensitivity in renal failure and advanced stages or other disorders that result in vascular calcification (incompressible arteries) [21, 33]. Our study excluded patients with ABI > 1.4, avoiding this bias and ABI was performed by the same operator during all study, reducing variability. Even with low sensitivity, it is an easy, low-cost, and non-invasive method for evaluating PAD, and its high specificity provides a good tool to evaluate clinical course of PAD. As our institution is a tertiary hospital reference for vascular disease and diabetes, our cohort presented a high prevalence of abnormal ABI, which led to a large sample to evaluate PAD progression, a strength in our work.

In our cohort, the diabetes duration was consistently associated with the presence and severity of PAD. Rare previous studies suggested that this illness duration should remain a risk factor even for patients who were still young when diagnosed [34]. The importance of this risk factor, associated with the high prevalence of abnormal ABI in recently diagnosed T2DM and drug-naive patients, confirms the existence of a delay in the detection of this disease reinforcing the benefit of an early investigation of PAD.

Our study found a decrease in HbA1c levels of patients who evolved with normalization or who maintained normal ABI values and also a correlation between final ABI and glycated hemoglobin variation. Kim et al. in a cohort study with a 6-year follow-up of 194 newly diagnosed T2DM patients found that the earlier achievement of glycemic control with HbA1c < 7% led to a lower frequency of macrovascular complications [17]. This relation is also described in T2DM patients (not drug-naive) in the UKPDS study, which presented that a reduction of 1% in HbA1c was associated with a 43% decrease in the risk of amputation or death [14]. In addition, a recent meta-analysis showed that DM patients with intensive glycemic control had 35% lower risk of non-traumatic lower limb amputation when compared to those with less intensive treatment strategies [35]. As we are aware, our study was the first to follow drug-naive T2DM patients since they were diagnosed and associate better glycemic control with ABI normalization.

In the Reduction of Atherothrombosis for Continued Health Registry (REACH Registry), the statin was associated with a 17% decrease in the occurrence of cardiovascular outcomes among individuals with PAD. Furthermore, other studies have suggested that the statin may reduce the incidence of lower-limb amputation and improve the distance covered by patients suffering from intermittent claudication [13, 14, 16, 28, 36, 37]. In our study, an improvement in the lipid profile and stabilization of the ABI was verified in drug-naive T2DM patients during the follow-up. In addition, it was associated with higher use of statins.

The association between PAD and albuminuria in patients in T2DM has been reported [38–40] A cross-sectional study with 206 T2DM patients found a correlation between ABI and microalbuminuria [38], agreeing with the Multi-Ethnic Study of Atherosclerosis (MESA), which demonstrated that T2DM patients with albuminuria had 1.90 higher risk of developing PAD [39]. The prevalence of albuminuria is high, even in newly diagnosed patients [41]. In a period of 5 years after diagnosis of T2DM, the cumulative incidence of albuminuria was found to be 17.3% [41]. In our study, albuminuria reduction was associated with an improvement of PAD evaluated by ABI. A better glycemic and lipid control have been described to impact both albuminuria and PAD progression. [13, 14]. It is more likely that these two factors caused a simultaneous improvement in albuminuria and stabilization of ABI and that albuminuria is only a risk marker for PAD.

The main limitation of our study was not stratifying physical exercise into mild, moderated and intense, which could have led to a more adequate analysis of real influence of physical activity on PAD. It occurred because we used ADA 2021 recommendations to classify exercise practice. Another weakness is the small number of patients in group 2 (drug-naive T2DM), but it occurred due to the difficulty of recruiting patients according to the strict inclusion criteria (T2DM with under three months duration and drug-naive). In addition, only 143 drug-naive patients have completed the follow-up so far, reducing our sample that was evaluated after the 3-year follow-up.

The ADA recommends that ABI test should be performed only in T2DM patients with signs or symptoms of PAD [10]. This is the principal guideline used in diabetic centers around the world and it underdiagnoses a lot of patients. Another guideline used is the ACC/AHA [18] It recommends screening with ABI in all patients with diabetes > 50 years or < 50 years with additional risk factors for cardiovascular disease. This criterion is more accurate than the ADA criterion, reducing the number of underdiagnosed patients, but it still fails in patients older than 50 years without any additional cardiovascular risk that present PAD. Our study has two principal conclusions: first, we followed type 2 diabetes mellitus patients for three years since the diagnosis and evaluated clinical progression of PAD using ABI, showing that albuminuria, glycemic, and lipid control could be related with ABI normalization. Additionally, our study suggests that ABI should be measured at diagnosis in T2DM patients, as we also observed in Felício et al. [11], indicating that current criteria to select patients to screen PAD with ABI must be simplified.

## Conclusion

Our cohort study is the first to follow T2DM patients for three years since diagnosis, evaluating clinical progression of PAD and using ABI, showing that albuminuria, glycemic, and lipid control could be related with ABI normalization. Additionally, our study suggests that ABI should be measured at diagnosis in T2DM patients, indicating that current criteria to select patients to screen PAD with ABI must be simplified.

## Data Availability

The datasets generated and/or analyzed during the current study are not publicly available due to privacy but are available from the corresponding author on reasonable request.

## Abbreviations

ABI: Ankle-brachial index
ACC: American college of cardiology
ADA: American diabetes association
AHA: American heart association
AUC: Area under the curve
BMI: Body mass index
DM: Diabetes mellitus
GFR: Glomerular filtration rate
HbA1c: Glycated hemoglobin
HDL-C: High-density lipoprotein cholesterol
LDL-C: Low-density lipoprotein cholesterol
PAD: Peripheral arterial disease
ROC: Receiver operating characteristic
T2DM: Type 2 diabetes mellitus
